# Host Phylogeny and Diet Shape Gut Microbial Communities Within Bamboo-Feeding Insects

**DOI:** 10.3389/fmicb.2021.633075

**Published:** 2021-06-22

**Authors:** Kuanguan Huang, Jie Wang, Junhao Huang, Shouke Zhang, Alfried P. Vogler, Quanquan Liu, Yongchun Li, Maowei Yang, You Li, Xuguo Zhou

**Affiliations:** ^1^Department of Forestry Protection, School of Forestry and Biotechnology, Zhejiang A&F University, Hangzhou, China; ^2^Department of Life Sciences, Natural History Museum, London, United Kingdom; ^3^Department of Life Sciences, Imperial College London Silwood Park, Ascot, United Kingdom; ^4^Department of Entomology, University of Kentucky, Lexington, KY, United States; ^5^State Key Laboratory of Subtropical Silviculture, Zhejiang A&F University, Hangzhou, China; ^6^Institute of Forestry Investigation and Planning of Guangning, Guangning, China; ^7^School of Forest Resources and Conservation, University of Florida, Gainesville, FL, United States

**Keywords:** cellulolytic bacteria, phylogeny, 16S rRNA sequencing, bamboo insects, gut microbiome

## Abstract

The gut microbiome plays an important role in a host’s development and adaption to its dietary niche. In this study, a group of bamboo-feeding insects are used to explore the potential role of the gut microbiota in the convergent adaptation to extreme diet specialization. Specifically, using a 16S rRNA marker and an Illumina sequencing platform, we profiled the microbial communities of 76 gut samples collected from nine bamboo-feeding insects, including both hemimetabolous (Orthoptera and Hemiptera) and holometabolous (Coleoptera and Lepidoptera) species, which are specialized in three distinct dietary niches: bamboo leaf, shoot, and sap. The gut microbiota of these insects were dominated by Proteobacteria, Firmicutes, and Bacteroidetes and were clustered into solid (leaf and shoot) and liquid (sap) dietary niches. The gut bacterial communities of insects feeding on solid diet overlapped significantly, even though these insects belong to phylogenetically distant lineages representing different orders. In addition, the presence of cellulolytic bacterial communities within the gut microbiota allows bamboo-feeding insects to adapt to a highly specialized, fiber-rich diet. Although both phylogeny and diet can impact the structure and composition of gut microbiomes, phylogeny is the primary driving force underlying the convergent adaptation to a highly specialized diet, especially when the related insect species harbor similar gut microbiomes and share the same dietary niche over evolutionary timescales. These combined findings lay the foundation for future research on how convergent feeding strategies impact the interplays between hosts and their gut microbiomes and how the gut microbiota may facilitate convergent evolution in phylogenetically distant species in adaptation to the shared diet.

## Introduction

The coevolution of animals and microbes within their guts facilitates their radiation into a wide variety of habitats (Muegge et al., 2011). In the long-term evolutionary process, they cooperate and interact, eventually forming an intimate symbiotic relationship. On the one hand, the community structure and the metabolic activities of gut microbes are affected by the gut microenvironment of the host animals (Douglas, 2015; Wang et al., 2017). On the other hand, gut microbes play a vital role in the food uptake and in providing nutrients (Brucker and Bordenstein, 2012; Scully et al., 2014; Handique et al., 2017).

In relying heavily on such coevolution with gut microbes, one remarkable consequence in animals is the occurrence of dietary specialization, which brings one of the important convergent evolutionary events and allows animals to adapt to various dietary niches (Dillon and Dillon, 2004). This is especially true with the phytophagous insect herbivores, where endogenous cellulases are usually lacking and cellulose digestion is, therefore, mediated/provided by the gut microorganisms (Martin, 1991). As the hosts, however, animals also affect the gut microbes morphologically, physiologically, and ecologically, contributing to the convergent evolution of the gut microbial community (Muegge et al., 2011; Delsuc et al., 2014; Baldo et al., 2017).

Recently, host diet and host phylogeny are identified as two important factors that modulate the microbiome composition and diversity in the animal gut (Behar et al., 2008; Ottesen and Leadbetter, 2011; Chen et al., 2016; Youngblut et al., 2019). Host food, touching directly the gut microorganisms, forms part of the microenvironment and can therefore render direct effects on the microbes. Previous researchers have suggested that altering a host’s diet could change the microbial community structure and related metabolic functions (Kane and Breznak, 1991; Broderick et al., 2004). The close association between phylogeny and the gut microbiota has also been found in mammals and insects (Delsuc et al., 2014; Sanders et al., 2014). Although, such a relationship is not difficult to draw since phylogeny is based on physical and genetic characteristics, which implies, to some extent, similar features such as gut morphology and metabolic system between close relatives and, thus, similar microenvironmental conditions. Consistent relationships have been reported where the relationship between host phylogeny and microbiota is similar to or even the same as that between diet and microbiota (Delsuc et al., 2014). However, inconsistent relationships were also found, and a dietary shift was often determined as the dominant driving factor of convergent evolution of the gut microbiota (Ley et al., 2008; Huang et al., 2021).

Many studies have been conducted and focus on the convergence of gut microbe assembly, but only a few of them are aimed at insects. Given that insects represent the most diverse and abundant animal clade with a wide variety of dietary niches (Behmer and Joern, 2008; Basset et al., 2012; Stork et al., 2015), a better understanding of the convergent evolution of the insect gut microbiota might help explain their successful colonization across the world. Bamboo-feeding insects represent one of the extreme examples of convergent herbivory (McKenney et al., 2018). Bamboos are fast-growing flowering plants distributed widely across East Asia, whose fiber is dominated by cellulose (Hao et al., 2018). Based on the structure and developmental stage of bamboos, these insects can be divided into leaf-, shoot-, and sap-specialized groups (Wang et al., 1998). A most recent study showed that convergent evolution of gut microbes in pandas, a mammal specialized in bamboo diet, was driven by diet rather than host phylogeny (Huang et al., 2021).

Here, we compared the gut microbiome structure and composition of nine bamboo-feeding insects using a high-throughput 16S ribosomal RNA (rRNA) sequencing on the Illumina platform. The sampled insects included both hemimetabolous and holometabolous species, which are specialized in three distinct dietary niches: bamboo leaf (Orthoptera and Lepidoptera), shoot (Coleoptera and Lepidoptera), and sap (Hemiptera). In this study, we addressed the following three questions: (1) Do these bamboo-feeding insects exhibit convergent evolution with their gut microbes? (2) If positive, which one, diet or phylogeny, is the primary driving force? Finally, (3) Does the diet or phylogeny impact the lignocellulosic gut microbes in these bamboo-feeding insects?

## Materials and Methods

### Sample Collection

We collected nine species of bamboo-feeding insects from Zhejiang (30°16′N–30°20′N, 119°28′E–119°36′E) and Guangdong (23°37′N, 112°21′E) provinces in China, including the larvae of *Crypsiptya coclesalis* Walker (CC) and *Sinibotys evenoralis* Walker (SE) and adults of *Ceracris nigricornis* Walker (CNM and CNF) feeding on leaves; larvae of *Apamea kumaso* Suqi (AK), *Melanotus cribricollis* (Faldermann) (MC), *Cyrtotrachelus buqueti* Guerin-Meneville (CB), and *Cyrtotrachelus thompsoni* Alonso-Zarazaga and Lyal (Coleoptera) (CT) feeding on shoots; and nymphs of *Hippotiscus dorsalis* (Stal) (HD) and adults of *Pseudoregma bambucicola* (Takahashi) (PB) feeding on sap (Supplementary Table 1). In total, 10 insect groups were included in this study since males and females of *Ce. nigricornis* were treated in separate groups due to the significant variations in the gut microbiota. Samples consisted of either a single individual for insects with a large body size (*Ce. nigricornis*, *Cy. thompsoni*, *Cy. buqueti*, and *H. dorsalis*) or two to five individuals for the smaller ones (*Cr. coclesalis*, *S. evenoralis*, *A. kumaso*, and *M. cribricollis*). For the bamboo wooly aphid, *P. bambucicola*, about 150 individuals were collected for one sample. All insects were collected with their specialized bamboo tissues in the field. Insect dissections were conducted within 24 h after the samples arrived in the laboratory. To confirm the morphological identification of insects and the possibility of cryptic species, the partial cytochrome oxidase subunit I (CO1) sequence was used as the DNA barcode for each individual or population, following Ye et al. (2017). Three nutrients of different bamboo dietary niches, i.e., leaf, shoot, and sap, were determined, including soluble protein (Coomassie brilliant blue staining G-250) (Zhang and Qu, 2003), soluble sugar (anthrone methods) (Zhang and Qu, 2003), and tannin (Folin–Denis assay) (Li et al., 2006).

### High-Throughput Illumina Sequencing

Samples were dissected using sterile instruments and techniques. The whole intestinal tract of each sample was dissected and homogenized in a biological sample homogenizer. The DNA of gut microbes was extracted using the QIAamp^®^ Fast DNA Stool Mini Kit (QIAGEN, Hilden, Germany). After DNA quality testing, the samples were sent for high-throughput Illumina sequencing. The V3–V4 regions of the 16S rRNA gene were amplified using primers 338F (5′ ACTCCTACGGGAGGCAGCA 3′) (McBain et al., 2003) and 806R (5′ GGACTACHVGGGTWTCTAAT 3′) (Ben Omar and Ampe, 2000). Each reaction contained 1.5 mM MgCl_2_, 0.4 μM each of deoxynucleoside triphosphate, 1.0 μM each of forward and reverse primers, 0.5 U of EX Taq^TM^ (TaKaRa, Dalian, China), 1 × PCR buffer, and 10 ng genomic DNA in PCR mixture (25 μl). The PCR thermal program included an initial denaturation at 94°C for 3 min, followed by 30 cycles of 94°C for 40 s, annealing at 56°C for 60 s, and extension at 72°C for 60 s, with a final extension at 72°C for 10 min. Mixed product from triplicate PCR reactions for each sample was used for further study. The PCR products were then checked with 1.0% agarose gel electrophoresis. The band of a correct size was excised and purified using TaKaRa MiniBEST Agarose Gel DNA Extraction Kit (TaKaRa, Dalian, China) and quantified with Nanodrop for equal molar amount from each sample. The sequencing samples were prepared using a TruSeq DNA Kit according to the manufacturer’s instruction. The purified library was diluted, denatured, re-diluted, mixed with PhiX (equal to 30% of the final DNA amount) as described in the Illumina library preparation protocols, and then applied to an Illumina MiSeq system for sequencing with the Reagent Kit v2 (2 × 250 bp). The sequence data have been submitted to the NCBI database under accession number DRA009676.

### Sequence Data Processing

We used the QIIME pipeline for the processing of the raw sequences (Caporaso et al., 2010). Paired-end reads were assembled using FLASH (Magoč and Salzberg, 2011). Reads with a quality score < 20, improper primers, and ambiguous characters were discarded. The resulting high-quality sequences were then clustered into 97% operational taxonomic units (OTUs) with the UPARSE algorithm (Edgar, 2013). Simultaneously, chimeras were checked and eliminated during clustering. Taxonomic classification of the OTUs were assigned with the rdp classifier (Wang et al., 2007). After discarding low-abundance OTUs (total counts of less than five), the resulting sequences of these OTUs from all the 76 samples were normalized by the trimmed mean of M values method using the BioConductor package EdgeR (Robinson et al., 2010). This method was chosen due to its sensitivity to address variations in the read counts of the OTUs among DNA sequencing libraries of widely different sizes in this study (McMurdie and Holmes, 2014). The resulting read counts of the OTUs after normalization were used for the following analysis regarding the overall gut bacterial diversity, while the relative abundance in the total microbiota of each insect group was used for tests on cellulolytic bacteria.

### Diversity Measurements and Statistical Analysis

Diversity indices, the number of observed OTUs, and the Shannon–Wiener index were used to compare the gut bacterial community composition among the different groups using the R package vegan (Faith, 1992; Oksanen et al., 2019). Significance tests of the bacterial community composition with analysis of similarities (ANOSIM) (Clarke, 1993) were carried out for the impact of host phylogeny and diet. Non-metric multidimensional scaling (NMDS) was used to analyze the differences in the bacterial community composition across orders and dietary niches based on the Bray–Curtis distance matrix (Lozupone and Knight, 2005). These distances were used to group the gut bacterial communities with the unweighted pair group method with arithmetic mean (UPGMA) algorithm. A ternary plot was presented to enrich the OTUs among the three bamboo diets (Kumar et al., 2017). These plots and calculations were performed using R v.3.6.0 (R Core Team, 2019). Canonical correlation analysis (CCA) (Hardoon et al., 2004) was performed using CANOCO software for Windows (Biometris, Wageningen, Netherlands) to evaluate the relationship between the gut microbiota composition and environment variables (Lourenço et al., 2018).

### Phylogenetic Analysis

Bacterial OTUs with putative cellulolytic activities were selected from the MiSeq sequencing data (Supplementary Table 2) according to previous studies on the identification of cellulolytic bacteria (Zhu et al., 2011; Bashir et al., 2013; Kong et al., 2014; Dantur et al., 2015; Manfredi et al., 2015) (Supplementary Tables 3–5). Bacterial sequences were aligned using MAFFT v7.471 (Katoh and Standley, 2013) and then imported into Gblocks v0.91b (Castresana, 2000) to exclude ambiguous regions. Insect cytochrome C oxidase subunit 1 (COI) sequences were aligned using the MUSCLE algorithm implemented in MEGA X (Kumar et al., 2018). Phylogenetic trees of both host insect and bacteria were conducted by maximum likelihood (ML) analyses using IQ-TREE v1.6.12 (Trifinopoulos et al., 2016) with the standard model selection (-m TEST option). Ultrafast bootstrap (UFBoot) approximation (Trifinopoulos et al., 2016) was specified with 5,000 replicates in a single run. In comparison to UFBoot support, the SH-aLRT branch test (Guindon et al., 2010) was also performed with 5,000 replicates. The tree of insects was rooted following a consensus phylogeny (Meusemann et al., 2010; Misof et al., 2014).

## Results

### Overall Gut Bacterial Community Among Bamboo-Feeding Insects

**FIGURE 1:**
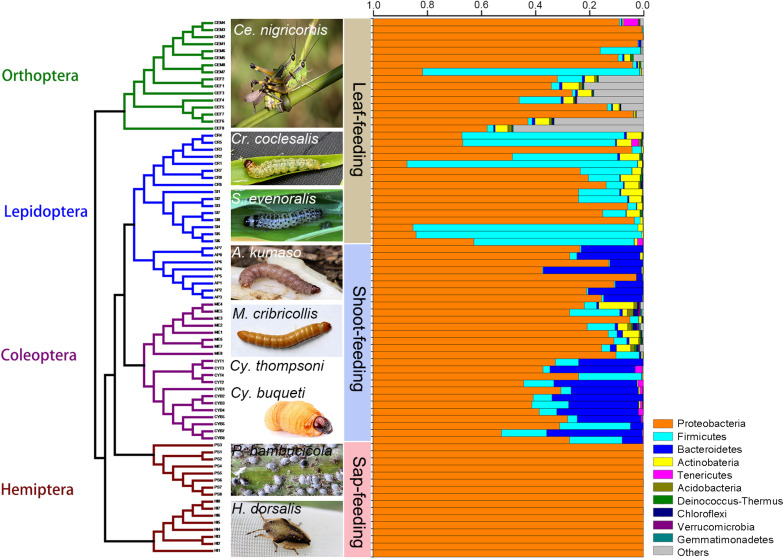
Phylogeny of bamboo-feeding insects with their corresponding gut microbiomes. The gut microbiota bacterial composition was estimated at the phylum level from a total of 76 gut samples. Insect orders are color-coded in the tree: *green*, Orthoptera; *blue*, Lepidoptera; *purple*, Coleoptera; *brown*, Hemiptera. The 10 most abundant bacterial phyla are shown in *different colored bars*.

The identification of insect species (Supplementary Table 1) was based on the morphology first and later confirmed by COI, a mitochondrial DNA marker (Figure 1). The microbial community compositions of 76 gut samples from 10 insect groups were obtained by MiSeq sequencing. A total of 10,085,555 high-quality sequence reads were identified as belonging to bacteria, with an average of 132,705 reads per sample (Supplementary Table 6). The average number of OTUs for each insect group was 325. Good’s coverage, which estimates the percentage of OTUs represented in an insect sample, averaged 99.9%, suggesting that the majority of the bacterial phylotypes in the insect gut were included in this study.

**FIGURE 2:**
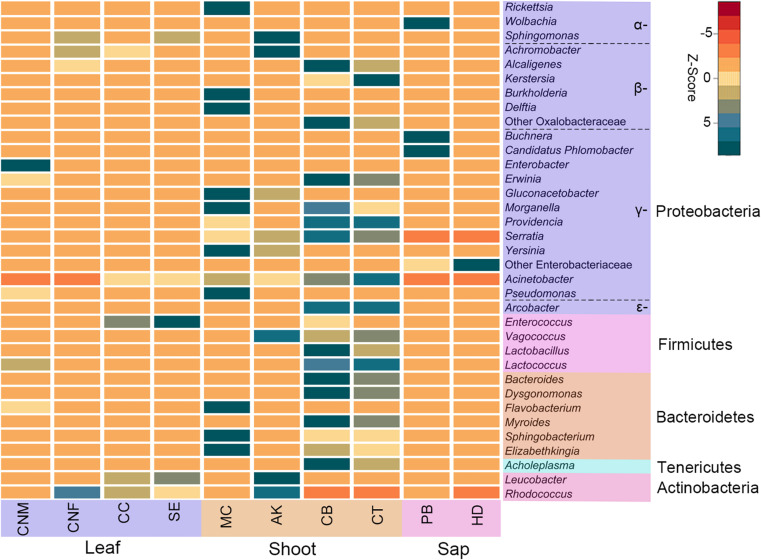
Heatmap of the top 35 most abundant bacterial genera in bamboo insect species. *Rows* are bacterial genera. *Columns* are the average abundance of insect samples within each group. Colors indicate a higher (*green*) or lower (*red*) abundance of bacterial genera in each insect group, identified by the *Z*-score. Bamboo-feeding insects include males and females of *Ceracris nigricornis* (*CNM* and *CNF*), *Crypsiptya coclesalis* (*CC*), *Sinibotys evenoralis* (*SE*), *Melanotus cribricollis* (*MC*), *Apamea kumaso* (*AK*), *Cyrtotrachelus buqueti* (*CB*), *Cyrtotrachelus thompsoni* (*CT*), *Pseudoregma bambucicola* (*PB*), and *Hippotiscus dorsalis* (*HD*).

The relative abundance of the microbial communities is shown in Figures 1, 2. The majority of sequences belongs to Proteobacteria (97.2% of the classified sequences), followed by Firmicutes (1.3%) and Bacteroidetes (1.2%) (Supplementary Figures 1, 2).

### Comparative Analysis of Gut Microbiota Across the Diets and Phylogeny

**FIGURE 3:**
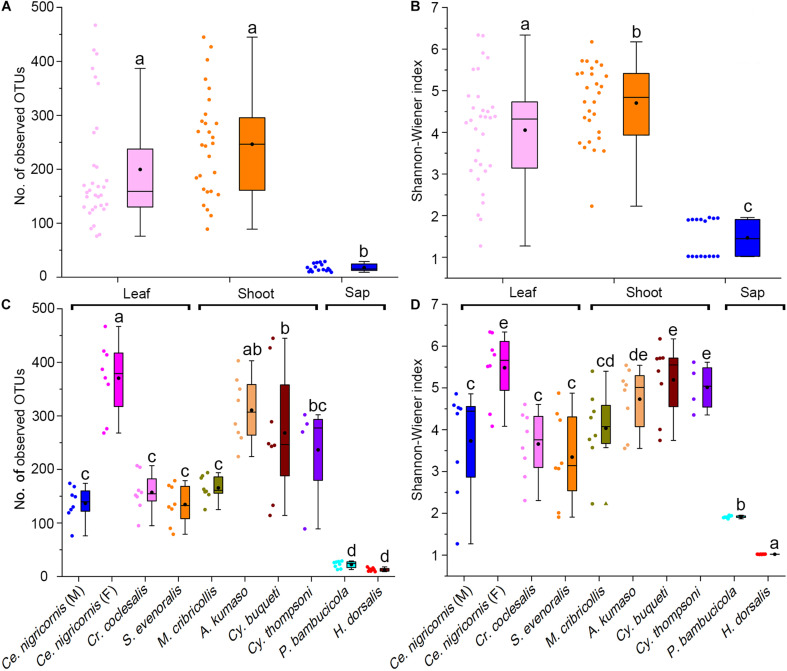
Alpha diversity of the gut bacterial community among bamboo-feeding insects. Number of observed OTUs of gut bacterial community among three bamboo diets **(A)** and host species **(C)**. Shannon-Wiener index of gut bacterial community among three bamboo diets **(B)** and host species **(D)**. Box plots representing variance within a particular sample were compared at both the phylogeny (insect species) and diet (dietary niches) levels. *Central square* is the mean, *central line* is the median, *box outline* equals 1 standard deviation (SD), *bar* denotes 1.5 SD, *colored dots* are samples, and *different letters on the box* indicate significant differences at *P* = 0.05 level.

**FIGURE 4:**
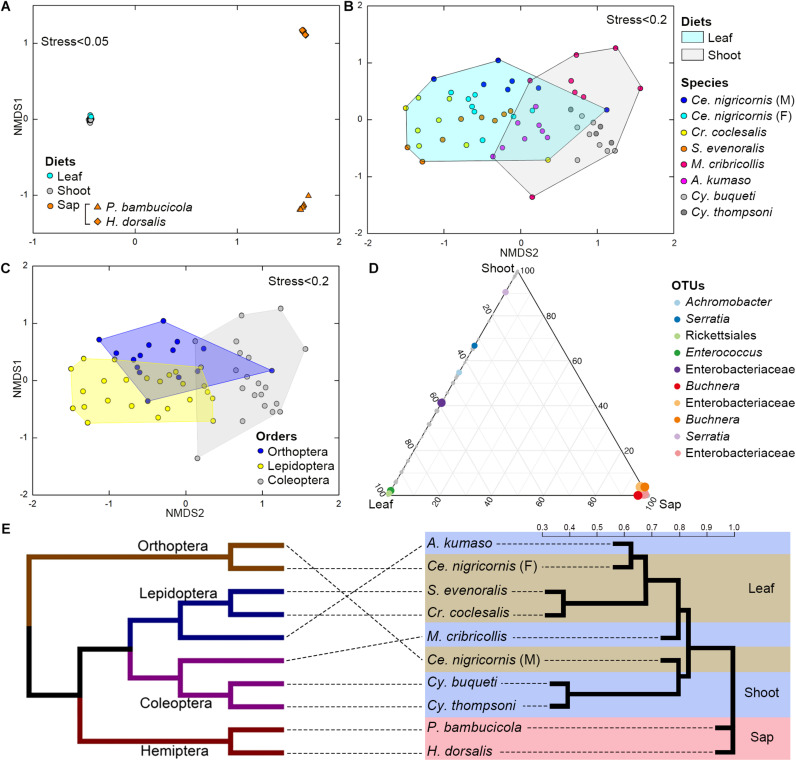
Impacts of diet and phylogeny on the gut bacterial communities among bamboo-feeding insects. **(A–C)** Non-metric multidimensional scaling (NMDS) analysis of the unweighted UniFrac distance matrix. Samples were colored by host diets **(A)**, host species **(B)**, and orders **(C)** of leaf-feeding and shoot-feeding species. *Distances between symbols* on the ordination plot reflect the relative dissimilarities in community memberships or structures. **(D)** Ternary plot of the operational taxonomic unit (OTU) distribution across three bamboo diets. Each *dot* represents one OTU, and the *size* and *position of each dot* represent the relative abundance and affiliation of the OTU with the different diets, respectively. *Colored dots* represent the top 10 enriched OTUs, whereas *gray dots* represent OTUs that are not significantly enriched in the three diets. **(E)** Cluster dendrogram of the gut bacterial communities comparing the host phylogeny. *Left*: The phylogenetic tree of the 10 insect groups constructed by COI sequences, which rooted according to Meusemann et al. (2010) and Misof et al. (2014). *Right*: The cluster dendrogram was analyzed based on the gut bacterial communities. *Shaded colors* represent different diets: *green*, bamboo leaf; *blue*, bamboo shoot; *red*, bamboo sap.

At the phylum level, the gut bacteria of the bamboo sap-feeding Hemiptera were dominated by Proteobacteria (>99.9%), the leaf-feeding Orthoptera and Lepidoptera were characterized by Proteobacteria (58.6–84.7%) and Firmicutes (4.3–35.2%), and the shoot-feeding Lepidoptera and Coleoptera were dominated by Proteobacteria (63.9–84.2%), Bacteroidetes (1.1–25.2%), and Firmicutes (0.7–16.5%) (Figures 1, 2). Thus, the gut bacteria of the three different diets showed remarkable differences, whereas within the same diet type, the community composition was similar. The results were consistent at the phylum level, order level, and genus level (Supplementary Figures 1, 2). All individuals within the diet class of leaf-feeding, shoot-feeding, and sap-feeding insects shared 58, 80, and 16 OTUs, respectively, out of a total of 1,015, 1,174, and 62 OTUs present in each feeding type (Supplementary Figure 3). Thirty-five OTUs were detected in all three feeding types. The alpha diversity indices (number of observed OTUs and the Shannon–Wiener index) of the gut bacteria in each of the two sap-feeding species were significantly lower than both leaf-feeding and shoot-feeding insects (Figures 3A–D). In addition, the Shannon–Wiener index of the leaf-feeding insects showed significantly higher values than those of the other two diets. The results of NMDS showed that the two species of sap-feeding insects grouped into separate clusters, while samples from the other two diets overlapped (Figures 4A–C). The bamboo diets had significant effects on the bacterial community composition (leaf vs. shoot vs. sap: *R* = 0.6885, *P* = 0.001). For the same diet type, the bacterial communities of the different insect species showed significant differences (CC vs. SE vs. CNM vs. CNF: *R* = 0.4209, *P* = 0.001; MC vs. AK vs. CT vs. CB: *R* = 0.6831, *P* = 0.001; PB vs. HD: *R* = 1.0000, *P* = 0.001), except for the closely related Lepidoptera CC vs. SE (*R* = −0.0039, *P* = 0.378) and the closely related Coleoptera CT vs. CB (*R* = −0.1507, *P* = 0.820) (Supplementary Table 7).

Among the insect orders, the microbiota showed significant differences in the ANOSIM test (Lepidoptera vs. Orthoptera vs. Coleoptera vs. Hemiptera: *R* = 0.6213, *P* = 0.001). The gut bacterial communities clustered by host diets, host species, and host orders (Figures 4A–C; Supplementary Figures 4A,B), and most variables contributed significantly to the gut community composition (Supplementary Table 7). Cluster results (Figure 4E) indicated that the bacterial communities of the sap-feeding Hemiptera formed a separate cluster, while the community structure of the rest of the insect species crossed in the dendrogram in both levels of host diet and orders.

**FIGURE 5:**
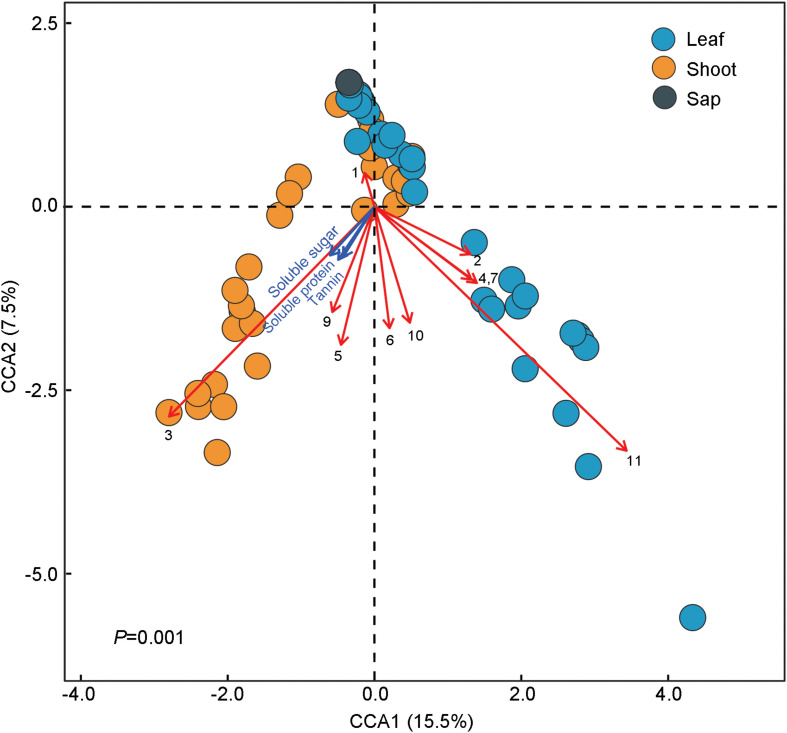
Correlations between the gut microbiota composition and dietary niches among bamboo-feeding insects. Canonical correlation analysis (CCA) was used to resolve the relationship between the gut microbiota composition and the three different bamboo diets. The *dots* refer to the microbiota composition at the operational taxonomic unit (OTU) level of each sample feeding on bamboo leaf, shoot, and sap, respectively. *Arrows* in the plot denote the magnitude and direction of the variable effects. *Black arrows* represent the top 10 most abundant phyla of gut microbiota: (1) Proteobacteria, (2) Firmicutes, (3) Bacteroidetes, (4) Actinobateria, (5) Tenericutes, (6) Acidobacteria, (7) Deinococcus–Thermus, (8) Chloroflexi, (9) Verrucomicrobia, (10) Gemmatimonadetes, and (11) Others. *Blue arrows* symbolize the three primary nutrients: soluble protein, soluble sugar, and tannin.

The ternary plot showed that four OTUs of Enterobacteriaceae, two OTUs of Rickettsiales and *Enterococcus* (Enterobacteriaceae), and one OTU of *Serratia* (Enterobacteriaceae) were significantly enriched in sap-, leaf-, and shoot-feeding insects (Figure 4D). Canonical correlation analyses showed that the bamboo diets explained 23.0% of the microbial community variation (CCA1 = 15.5%, CCA2 = 7.5%) (Figure 5). All the three nutritional compounds—soluble sugar, soluble protein, and tannin—were significantly linked to the microbial community changes of Bacteroidetes, Verrucomicrobia, Tenericutes, Acidobacteria, and Gemmatimonadetes in insect gut with different bamboo diets (*P* = 0.001).

### Comparative Analysis of Putative Cellulolytic Bacteria Along the Diets and Phylogeny

**FIGURE 6:**
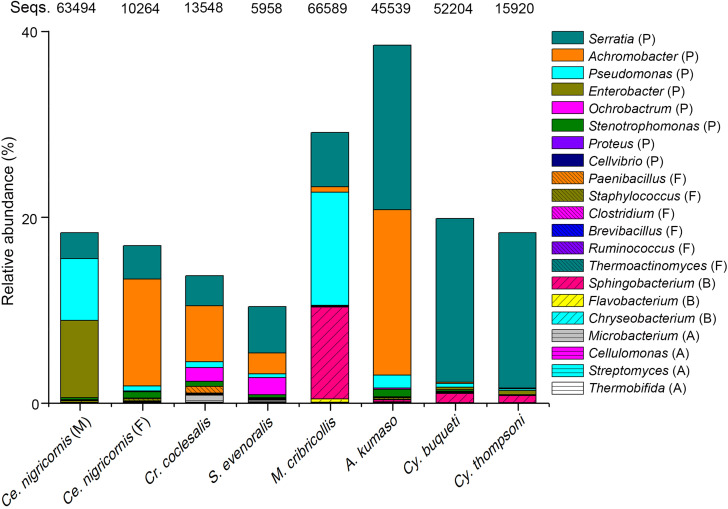
Relative abundance of the putative cellulolytic bacterial sequences among the bamboo-feeding insects. Abbreviations of bacterial phyla: *P*., Protebacteria; *F*., Firmicutes; *B*., Bacteroides; *A*., Acidobacteria; and *Seqs*, number of sequences.

**FIGURE 7:**
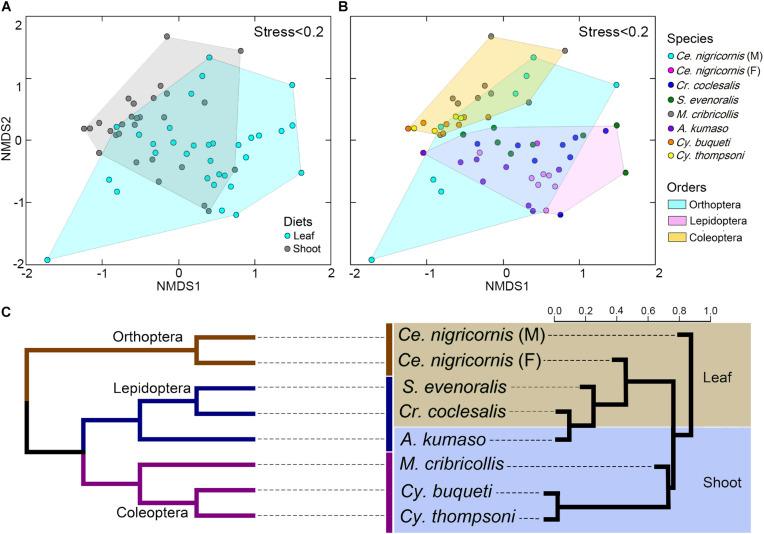
Impacts of diet and phylogeny on the gut cellulolytic bacterial communities among bamboo-feeding insects. **(A,B)** Non-metric multidimensional scaling (NMDS) analysis of the unweighted UniFrac distance matrix. Samples were colored by host diets **(A)** and host species and orders **(B)**. *Distances between symbols* on the ordination plot reflect the relative dissimilarities in community memberships or structures. **(C)** Cluster dendrogram comparing the host phylogeny. *Left*: The phylogenetic tree of the eight leaf- and shoot-feeding insect groups was constructed by COI sequences, which rooted according to Meusemann et al. (2010) and Misof et al. (2014). *Right*: The cluster dendrogram was analyzed based on the gut cellulolytic bacterial communities. *Shaded colors* represent different diets: *green*, bamboo leaf; *blue*, bamboo shoot.

Many bacterial genera are known to exhibit cellulose-degrading abilities (Supplementary Tables 3–5). A total of 131 bacterial OTUs detected in the total 2,752 OTUs of this study could be assigned to these potential cellulolytic bacteria (Figure 6, Supplementary Table 2, and Supplementary Figure 5). Almost all cellulolytic bacteria were from the eight groups of leaf- and shoot-feeding insects (Supplementary Table 2), but they were largely missing in the bamboo sap-feeding insects. The majority of cellulolytic microbes were members of Proteobacteria (41 OTUs, 21.30% of the total 1,133,399 sequences of all the gut bacteria in these eight insect groups), Bacteroidetes (51 OTUs, 2.64%), Firmicutes (32 OTUs, 0.13%), and Actinobacteria (7 OTUs, 0.06%) (Figure 6 and Supplementary Figure 5). However, the cellulolytic bacterial community of the two diets showed significant differences (leaf vs. shoot: *R* = 0.2442, *P* = 0.001) (Figure 7 and Supplementary Table 7). Within the same diet class, there were differences in the community composition between distantly related species (CC vs. SE vs. CNM vs. CNF: *R* = 0.4267, *P* = 0.001; MC vs. AK vs. CT vs. CB: *R* = 0.4502, *P* = 0.001), but not in the two pairs of the close relatives CC vs. SE (*R* = −0.0151, *P* = 0.464) and CT vs. CB (*R* = −0.1287, *P* = 0.806). The three insect orders also showed significant differences (Lepidoptera vs. Orthoptera vs. Coleoptera: *R* = 0.3425, *P* = 0.001). Cluster analysis of cellulolytic bacteria (Figure 7C) indicated that the functional bacterial community structure was most closely correlated with host phylogeny rather than host diet. The cellulolytic bacterial communities of Lepidoptera and Coleoptera clustered separately in the dendrogram, while Orthoptera clustered distantly from the rest of the six insect groups.

## Discussion

### Gut Microbiomes of Bamboo-Feeding Insects

The gut bacterial community of the bamboo-feeding insect herbivores is rich and diverse compared to other insect herbivores and bamboo-feeding mammals (Yun et al., 2014; Kim et al., 2017; Huang et al., 2021). Proteobacteria presented the great majority of the bacteria in the gut of sap-feeding bamboo insects, whereas Proteobacteria was the most abundant phylum, in addition to a considerable proportion of Firmicutes in both leaf- and shoot-feeding bamboo insects. Meanwhile, Bacteroidetes was found as the second abundant phylum in shoot-feeding insects. Proteobacteria, Firmicutes, and Bacteroidetes are usually found as the predominant phyla in the gut of other insects and mammals (Ley et al., 2008; Chen et al., 2018), including bamboo-eating giant panda and red panda (Zhu et al., 2011; Kong et al., 2014).

Comparative analysis showed that both diet (dietary niche) and phylogeny (i.e., insect species and order) significantly affected the gut bacterial community structure among bamboo-feeding insects (Figures 3, 4, Supplementary Figure 4, and Supplementary Table 7). The result is consistent with previous studies showing that diet and taxonomy influenced the insect gut microbiota (Colman et al., 2012; Liu Y. et al., 2019). The gut morphology and physicochemical conditions can vary substantially among insects and developmental stages (Engel and Moran, 2013; Zhao et al., 2018), which can influence the community structure of host-specific gut microbiota (Yun et al., 2014). In this study, Coleoptera (shoot-feeding and larva), Orthoptera (leaf-feeding and adult), and Lepidoptera (shoot- or leaf-feeding, larva) share a chewing mouthpart and a similar gut structure, while Hemiptera (bamboo sap-feeding insects, nymph, and adult) exhibit sucking mouthparts and a dramatically different gut anatomy without producing a peritrophic matrix (Lehane, 1997). The results indicated that the gut bacteria in the sap-feeding insects were significantly different from both the leaf- and shoot-feeding groups (Figures 3, 4, Supplementary Figure 4, and Supplementary Table 7). A previous study found that the gut bacterial diversity of omnivorous insects was significantly higher than that of carnivores and herbivores as omnivores consumed more diverse foods than do carnivores and herbivores (Yun et al., 2014). Microbial symbionts have been shown to take on various functions benefiting their hosts, such as nutritional upgrading of the diet (Douglas, 1998; Akman et al., 2002). Given that leaves and shoots are more complex than sap, structurally and chemically speaking, more diverse microbes might participate in the digestion and absorption of these types of food, in line with other studies that linked the higher bacterial diversity with the type of consumed food (Anderson et al., 2013).

### Diet and Phylogeny Structure the Gut Bacterial Community

The gut bacterial communities were found not simply to be the result of convergent evolution with diet or phylogeny, although both the host diet and phylogeny had an important effect on the diversity of gut bacterial communities. These herbivorous insects were clearly clustered into two types of bamboo diets, i.e., liquid-feeding (sap) with sucking mouthparts and solid-feeding (leaf and shoot) with chewing mouthparts (Figure 4E). Although the bamboo diets explained a considerable proportion of the microbial community variation in the CCA (Figure 5), the gut bacterial structure of those solid-feeding insects overlapped largely, even when these insect groups are phylogenetically divergent (Figures 4A–C,E). The result was consistent with previous studies which found that the diet and phylogeny structured the gut bacterial communities, while the primary driving force depends on the different animal groups (Colman et al., 2012; Delsuc et al., 2014; Amato et al., 2019). Studies on higher termites and longhorn beetles showed that diet was the primary determinant of the gut bacterial community structure (Mikaelyan et al., 2015; Kim et al., 2017). The gut microbial communities of xylophagous termites were significantly similar to each other, while detritivorous termites were significantly similar to other detritivorous insects in the distantly related orders Coleoptera and Diptera (Colman et al., 2012). In mammals, diet adaptation was considered to be the main driving factor affecting the microbial community structure (Ley et al., 2008; Faith et al., 2011; Muegge et al., 2011; Delsuc et al., 2014), although two recent studies suggested that the impact of host phylogeny and physiology outweighs that of host dietary niche in gut microbiome divergence (Nishida and Ochman, 2018; Amato et al., 2019). Furthermore, a recent study on bamboo-feeding pandas confirmed that the specialized herbivorous diet rather than the host phylogeny was the dominant driver of gut microbiome convergence within beers (Arctoidea) (Huang et al., 2021).

Additionally, this study also showed that the microbiome community composition did not show differences in the phylogenetically closely related host species when the insects were feeding on the same bamboo tissues (Figure 4E). Bamboo borers (Lepidoptera: Crambidae: Pyraustinae) and weevils (Coleoptera: Curculionidae: Dryophthorinae) from the same subfamily shared similar gut microbiota when they have the same diet, while significant differences were found between leaf-feeding Lepidoptera and Orthoptera and shoot-feeding Coleoptera and Lepidoptera (Supplementary Figure 4 and Supplementary Table 7). This result complements previous works on specialized bamboo-feeding mammals from two distant orders (McKenney et al., 2018). For monophagous insects, diet is usually consistent within species, such as the bamboo-feeding insects in this study. Strikingly, the gut flora of male and female grasshoppers of the same species (Orthoptera) showed significant variations, which may be explained by their significant differences in food consumption (Xu, 1984). The variations between the sexes have been found in the adults of silkworm (Chen et al., 2018) and fruit flies (Gujjar et al., 2017). Meanwhile, species and diet might affect the gut flora in different directions in polyphagous species. Studies of the gut bacterial communities of fungal farming ants (Tribe: Attini) and pine processionary moth larva (*Thaumetopoea pytiocampa*) suggest that different diets impacted the microbiota diversity within the insect species (Aylward et al., 2014; Strano et al., 2018).

We determined three important nutrition compounds (soluble protein, soluble sugar, and tannin) of each bamboo diet in order to evaluate whether they influenced the insect gut bacterial composition. The results indicated that five of the 10 most abundant phyla of the bacterial community showed positive correlations with these nutrients. Plant nutrients significantly impact insect fitness, including their symbiotic bacteria that help insect herbivores to efficiently use plant diets that are limited in essential nitrogen (Hansen and Moran, 2014). Meanwhile, tannin production has been recognized as a plant defense against herbivores (Coley et al., 1985; Bernays et al., 1989). Anyway, we are just beginning to understand the role of diet–microbe interactions in insect herbivores, although dietary influences (nutrients) on rodent and human microbiota have been applied in dietary interventions to change the composition of the gut bacteria for a healthier homeostatic balance (Kolodziejczyk et al., 2019).

### Phylogeny Is the Primary Driving Force of Cellulolytic Bacterial Communities

Due to the unique characteristics of pandas feeding on bamboo, comparative studies were carried out to confirm the convergent evolution between the giant and red pandas (Hu et al., 2017). However, the results indicated that the pandas were adapted to the specialized bamboo diet with the presence of cellulose-digesting bacteria, but still harbor a carnivore-like gut microbiota (Xue et al., 2015). Recent studies on the isolation of cellulolytic bacteria from the intestine of termites and caterpillars supported the findings of the insects’ capacity to degrade lignocellulose (Bashir et al., 2013; Dantur et al., 2015; Manfredi et al., 2015). Interestingly, we found 131 OTUs (Figure 6 and Supplementary Table 2) corresponding to genera known to be cellulolytic in pandas and these insects, which are likely to function in cellulose degradation also in the bamboo-feeding insects. ANOSIM indicated that all the factors including the diet, species, and order showed significant impacts, while the closely related bamboo borers and weevils under the same diet shared, respectively, similar cellulolytic bacterial communities (Supplementary Table 7). Further analysis by cluster suggested that the insect gut functional bacterial community structure was mainly affected by phylogeny in their adaptive radiation for different ecological niches.

Although the diet types and phylogenetic groups in this study overlapped for Hemiptera, Orthoptera, and Coleoptera due to the biology of the bamboo-feeding insects, both leaf- and shoot-feeding species of Lepidoptera were included in this study. The bamboo tissue consumed by Hemiptera and Orthoptera was limited, respectively, to sap and leaf, while species of Coleoptera and Lepidoptera were found feeding on both bamboo leaf and shoot. Due to the rare occurrence of Coleoptera species feeding on bamboo leaf, these species were not able to be analyzed, which would greatly support in fully addressing the questions raised in this study. However, the results from the cellulolytic bacterial community indicated that the shoot-feeding *A. kumaso* (Lepidoptera) clustered with the leaf-feeding Lepidoptera rather than the shoot-feeding Coleoptera. These data imply that the ability of cellulolytic digestion evolves readily in different lineages of extant insect through the adoption of a specialized microbiome. These bacterial communities are critical for efficient lignocellulose metabolism and, thus, survival, as shown in xylophagous termites, which required a specialized gut microbial community (Breznak and Brune, 1994; Lazuka et al., 2018; Liu N. et al., 2019). The putative cellulolytic bacterial communities in this study are essential for the nutrition acquisition of insect hosts. It is also worth noting that the potential cellulose degradation ability was inferred by phylogenetic analysis with the cellulolytic bacteria from pandas and other insects, which clearly placed the OTUs detected in bamboo insects into known cellulolytic clades, suggesting that host phylogeny is a major driving factor of convergence in gut microbiome compositions and functional communities.

## Summary and Perspectives

Using a 16S rRNA marker and an Illumina sequencing platform, we investigated the impacts of host phylogeny and dietary niche on the gut microbiota structure and composition of bamboo-feeding insects across highly divergent lineages. The presence of cellulolytic bacterial communities within the gut microbiota allows bamboo-feeding insects to adapt to a highly specialized, fiber-rich diet. Although both phylogeny and diet can impact the structure and composition of gut microbiomes, phylogeny is the primary driving force underlying the convergent adaptation to a specialized diet, especially when the related insect species harbor similar gut microbiomes and share the same dietary niche over evolutionary timescales. These findings laid the foundation for understanding how convergent feeding strategies impact the host–microbiome relationships and how the gut microbiota may facilitate convergent evolution in phylogenetically divergent animals in the adaptation to the shared bamboo diet.

## Data Availability Statement

The datasets presented in this study can be found in online repositories. The names of the repository/repositories and accession number(s) can be found below: https://www.ddbj.nig.ac.jp/, PRJD89344.

## Author Contributions

KH and JW carried out the experiments, analyzed the data, and drafted the manuscript. SZ, AV, QL, YnL, and YuL revised the manuscript. MY collected and analyzed the data. JH designed the research, revised the manuscript, and received the funding. XZ oversaw the entire project, and revised and edited the manuscript. All authors have read and approved the final submitted version.

## Conflict of Interest

The authors declare that the research was conducted in the absence of any commercial or financial relationships that could be construed as a potential conflict of interest.
