# Supplementary material for: Host Phylogeny and Diet Shape Gut Microbial Communities Within Bamboo-Feeding Insects
Source: Front Microbiol. 2021 Jun 22;12:633075. doi: 10.3389/fmicb.2021.633075 (PMC8260032; doi:10.3389/fmicb.2021.633075)
Supplement: Supplementary file 1 [file Data_Sheet_1.docx]

Supplementary Materials

Host phylogeny and diet shape gut microbial communities within bamboo-feeding insects

Kuanguan Huang^1†^, Jie Wang^1†^, Junhao Huang^1^*, Shouke Zhang^1^, Alfried P. Vogler^2,3^, Quanquan Liu^4^, Yongchun Li^5^, Maowei Yang^6^, You Li^7^, Xuguo Zhou^4^

*^1^Department of Forestry Protection, School of Forestry and Biotechnology, Zhejiang A &F University, Hangzhou, China*

*^2^Department of Life Sciences, Natural History Museum, London, UK*

*^3^Department of Life Sciences, Silwood Park Campus, Imperial College London, Ascot, UK*

*^4^Department of Entomology, University of Kentucky, Lexington, KY, 40546, USA*

*^5^ State Key Laboratory of Subtropical Silviculture, Zhejiang A&F University, Hangzhou, China*

*^6^Institute of Forestry Investigation and Planning of Guangning, Guangning, China*

*^7^School of Forest Resources and Conservation, University of Florida, Gainesville, USA*

----------------------------------------------------------------------------------------------------------------

† These authors contributed equally to this study

*** Correspondence:**

Junhao Huang: [huangjh@zafu.edu.cn](mailto:huangjh@zafu.edu.cn)

**Running title**

Phylogeny and diet shape insect gut microbiomes

Supplementary Figures


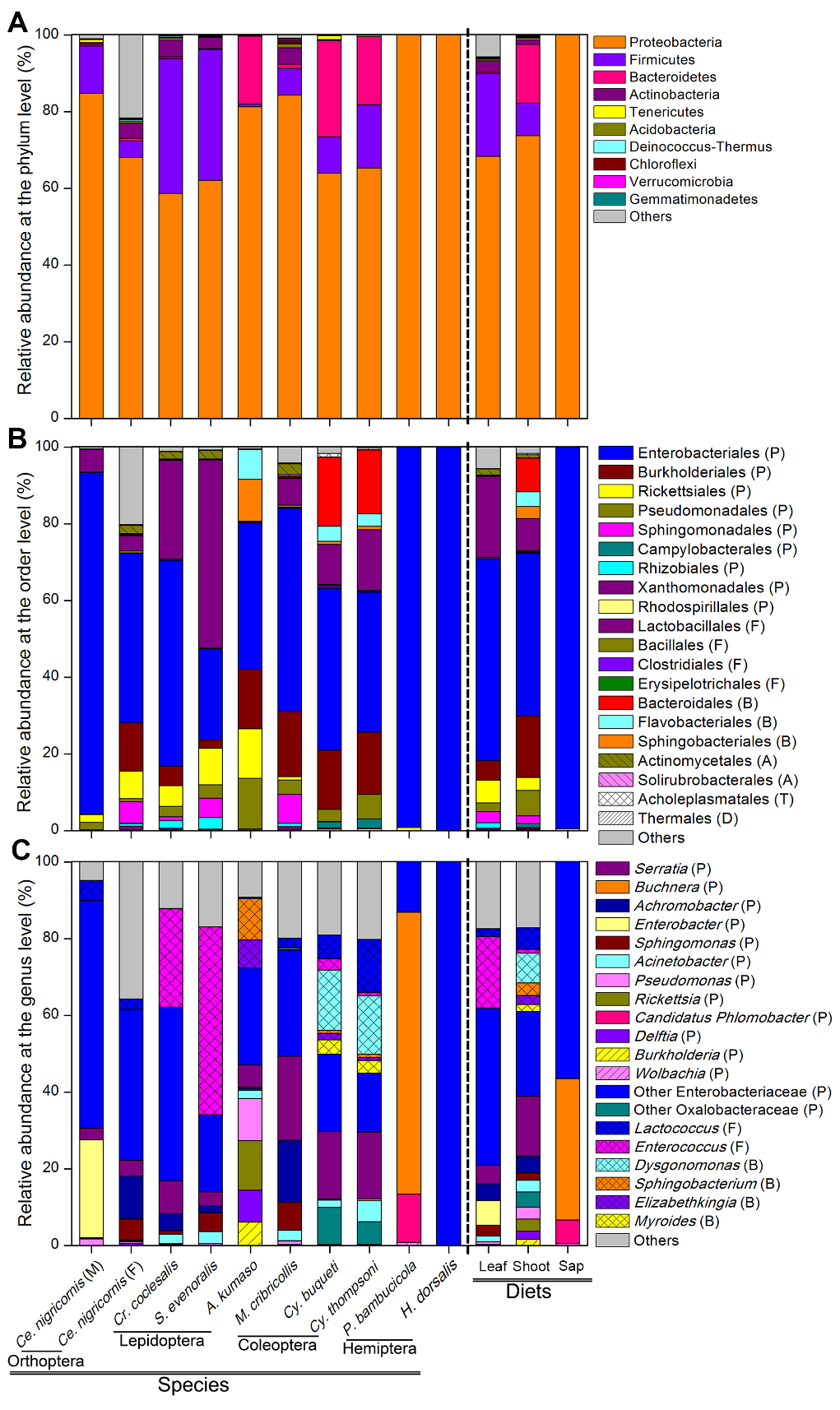


**Figure S1**


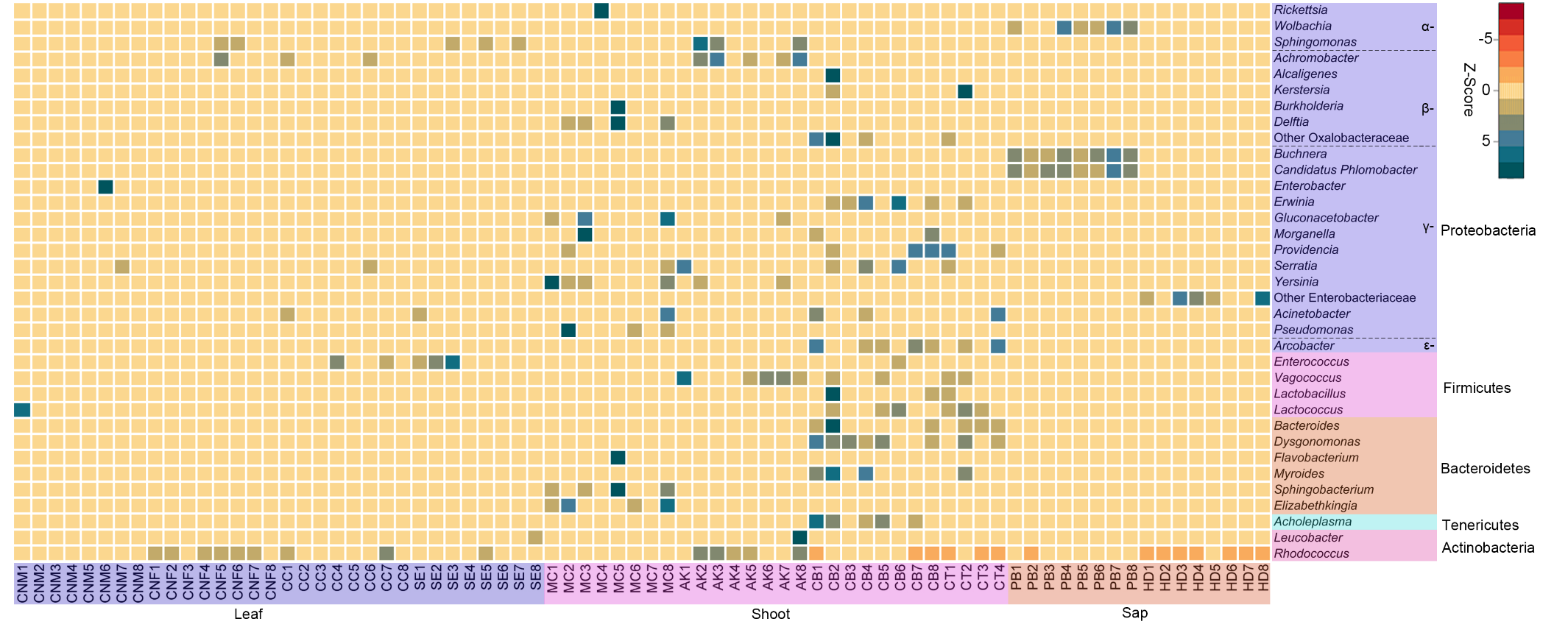


**Figure S2**


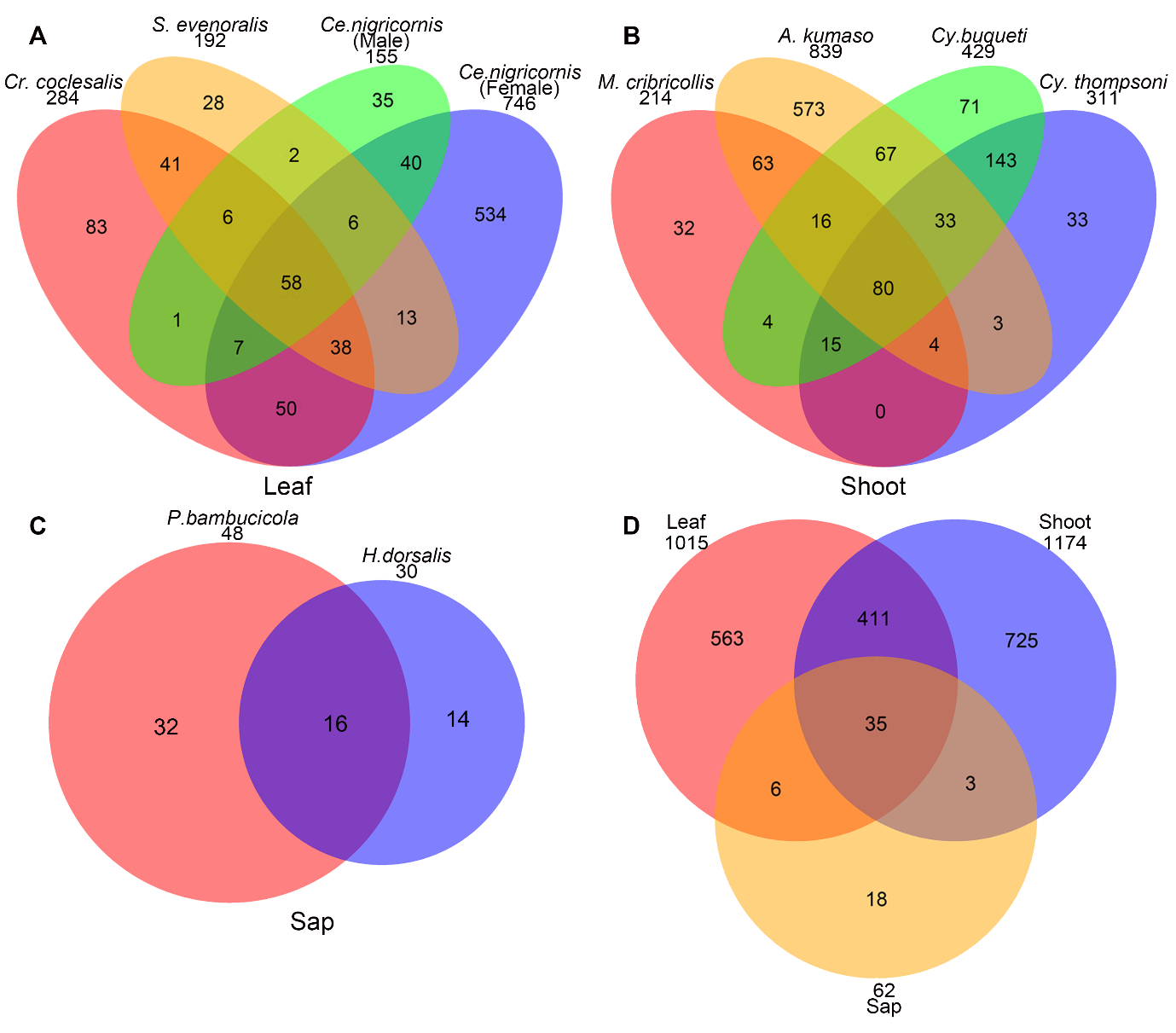


**Figure S3**


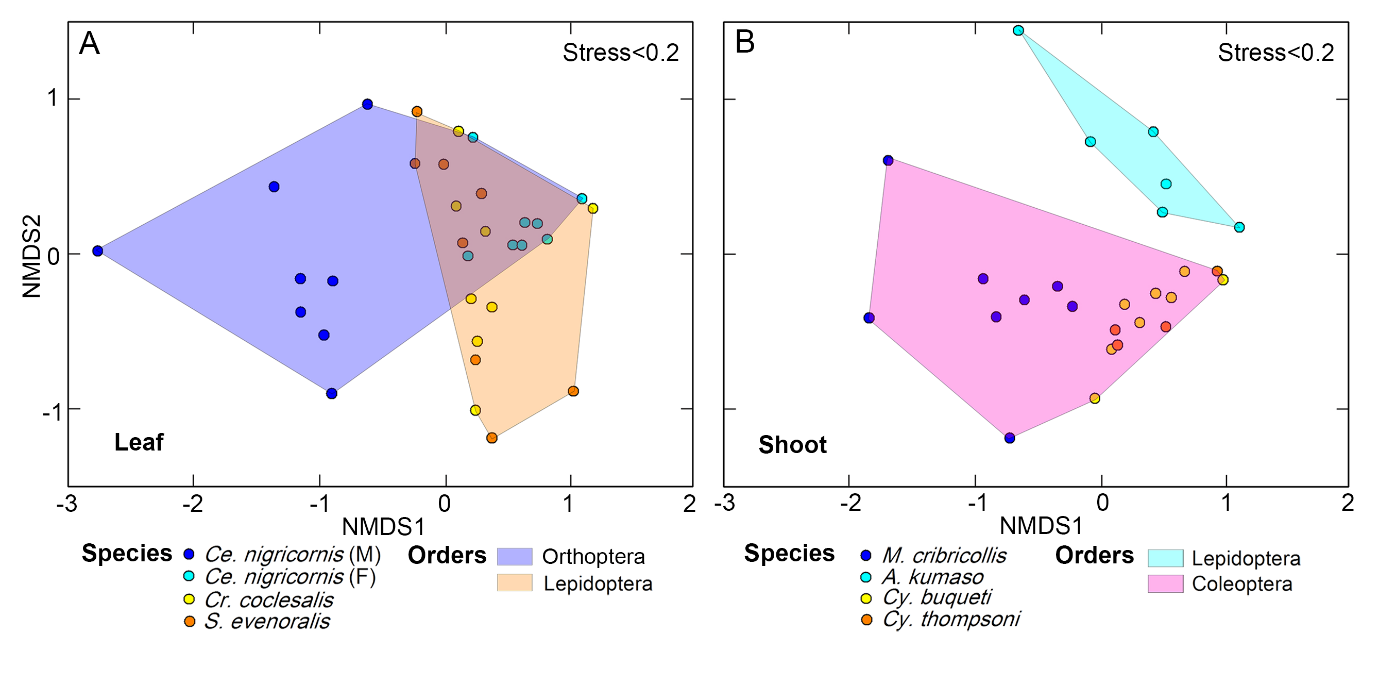


**Figure S4**


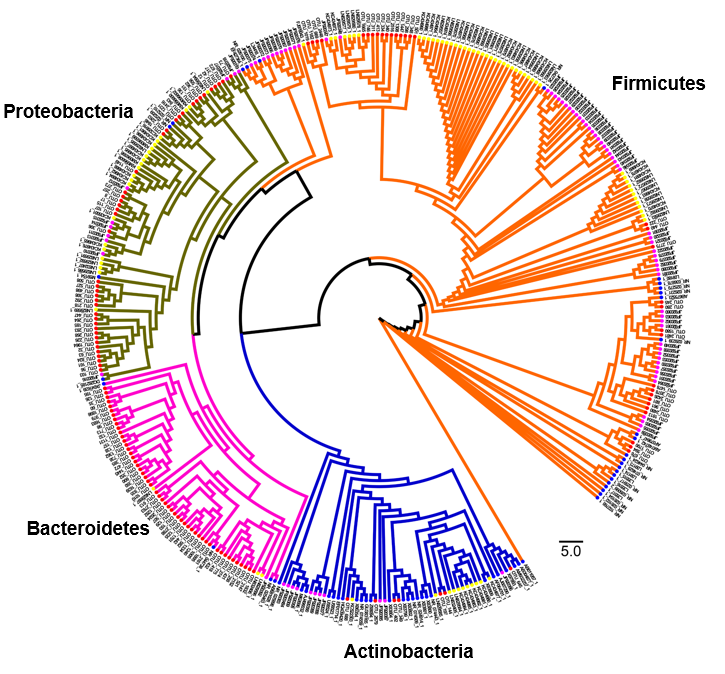


**Figure S5**

**Figure Legends**

**Figure S1. Gut bacterial community composition among bamboo-feeding insects.** (a) phylum level; (b) order level; (c) genus level. Bacterial phyla included were: P. Protebacteria, F. Firmicutes, B. Bacteroides, A. Actinobacteria, T. Tenericutes, D. Deinococcus-Thermus.

**Figure S2.** **Heatmap of the top 35 most abundant bacterial genera in bamboo insect samples**. Rows are bacterial genera. Columns are insect samples. Colors indicate a higher (green) or lower (red) relative abundance in each sample, identified by Z-score. Bamboo-feeding insects included here are male and female of *Ceracris nigricornis* (CNM & CNF), *Crypsiptya coclesalis* (CC), *Sinibotys evenoralis* (SE), *Melanotus cribricollis* (MC), *Apamea kumaso* (AK), *Cyrtotrachelus buqueti* (CB), *Cyrtotrachelus thompsoni* (CT), *Pseudoregma bambucicola* (PB), and *Hippotiscus dorsalis* (HD).

**Figure S3.** **Venn diagram comparing the shared OTU numbers in gut microbiome of bamboo-feeding insects.** These bamboo-feeding insects were grouped following three different dietary niches, i. e. bamboo leaf, shoot and sap. Specifically, leaf-feeding species included *Cr. coclesalis*, *S. evenoralis*, and male and female of *Ce. nigricornis* (A); shoot-feeding species involved *A. kumaso*, *M. cribricollis*, *Cy. thompsoni* and *Cy. buqueti* (B); and sap-feeding species were comprised of *P. bambucicola* and *H. dorsalis* (C). The overall comparison of the three dietary niches was provided in D.

**Figure S4.** **Impact of phylogeny on gut bacterial communities within the same bamboo dietary niche.** Non-Metric Multi-Dimensional Scaling (NMDS) analysis of the unweighted UniFrac distance matrix. Distances between symbols on the ordination plot reflect relative dissimilarities in community memberships or structures. Samples were colored by host species and host orders of bamboo leaf-feeding (A) and shoot-feeding (B) groups.

**Figure S5. Phylogenetic analysis of the putative cellulolytic OTUs in this study with known cellulolytic bacteria.** The maximum likelihood method was used to for this analysis. Branch colors: orange, Firmicutes; blue, Actinobacteria; green, Proteobacteria; purple, Bacteroidetes. Dot colors: red, OTUs in this study (Table S2); blue, Kong et al., 2014 (Table S3); purple, from Zhu et al., 2011 (Table S4); yellow, from Dantur et al., 2015, Bashir et al., 2013 and Manfredi et al., 2015 (Table S5).

**Table S1. Information of the bamboo-feeding insects collected in this study**

| **Insect species** | **Taxonomic status** | **ID** | **N^1^** | **Host plant** | **Diet** | **Collection site** | **GPS^2^** | **Collection date** | **Stage^3^** |
| --- | --- | --- | --- | --- | --- | --- | --- | --- | --- |
| *Crypsiptya coclesalis* Walker | Lepidoptera:Pyralidae | CC | 8 | *Phyllostachys violascens* | Leaf | Lin'an, Zhejiang | 30°20'N,119°28'E | 2017.7.29 | Larva |
| *Sinibotys evenoralis* Walker | Lepidoptera:Pyralidae | SE | 8 | *P. violascens* | Leaf | Lin'an, Zhejiang | 30°20'N,119°28'E | 2017.7.29 | Larva |
| *Ceracris nigricornis* Walker  *Ceracris nigricornis* Walker | Orthoptera:Oedipodidae  Orthoptera:Oedipodidae | CNM | 8 | *P. violascens*  *P. violascens* | Leaf  Leaf | Lin'an, Zhejiang  Lin'an, Zhejiang | 30°20'N,119°28'E | 2017.10.28  2017.10.28 | Male |
|  |  | CNF | 8 |  |  |  | 30°20'N,119°28'E |  | Female |
| *Melanotus cribricollis* (Faldermann) | Coleoptera:Elateridae | MC | 8 | *P. dulcis* | Shoot | Lin'an, Zhejiang | 30°16'N,119°36'E | 2017.4.17 | Larva |
| *Apamea kumaso* Suqi | Lepidoptera:Noctuidae | AK | 8 | *P. dulcis* | Shoot | Lin'an, Zhejiang | 30°16'N,119°36'E | 2017.4.29 | Larva |
| *Cyrtotrachelus buqueti* Guerin-Meneville | Coleoptera:Curculionidae | CB | 8 | *Bambusa textilis* | Shoot | Zhaoqing, Guangdong | 23°37'N,112°21'E | 2017.8.13 | Larva |
| *Cyrtotrachelus thompsoni* Alonso-Zarazaga et Lyal | Coleoptera:Curculionidae | CT | 4 | *B. textilis* | Shoot | Zhaoqing, Guangdong | 23°37'N,112°21'E | 2017.8.13 | Larva |
| *Pseudoregma bambucicola* (Takahashi) | Hemiptera:Hormaphididae | PB | 8 | *B. multiplex* | Sap | Lin'an, Zhejiang | 30°15'N,119°44'E | 2018.6.25 | Adult |
| *Hippotiscus dorsalis* (Stal) | Hemiptera:Pentatomidae | HD | 8 | *P. violascens* | Sap | Lin'an, Zhejiang | 30°20'N,119°28'E | 2018.5.17 | Nymph |

“^1^”: Number of samples for each insect species.

“^2^”: GPS coordinates are denoted by latitude and longitude.

“^3^”: Developmental stage.

**Table S2. 16S rRNA sequences of putative cellulolytic bacteria in bamboo-feeding insects**

| **Phylogeny** | **Genus** | **OTU^1^** |
| --- | --- | --- |
| Phylum Firmicutes, Class Clostridia, Order Clostridiales | | |
| Fam.Ruminococcaceae | *Ruminococcus* | OTU_1700 |
|  |  | OTU_594 |
|  |  | OTU_676 |
| Fam.Clostridiaceae | *Clostridium* | OTU_2480 |
|  |  | OTU_2078 |
|  |  | OTU_280 |
|  |  | OTU_2426 |
|  |  | OTU_1478 |
|  |  | OTU_1813 |
|  |  | OTU_2481 |
|  |  | OTU_1580 |
|  |  | OTU_245 |
|  |  | OTU_961 |
|  |  | OTU_881 |
| Phylum Firmicutes,Class Bacilli, Order Bacillales | | |
| Fam.Thermoactinomycetaceae | *Thermoactinomyces* | OTU_2775 |
| Fam.Paenibacillaceae | *Brevibacillus* | OTU_501 |
| Fam.Staphylococcaceae | *Staphylococcus* | OTU_222 |
|  |  | OTU_440 |
| Fam.Paenibacillaceae | *Paenibacillus* | OTU_334 |
|  |  | OTU_1665 |
|  |  | OTU_669 |
|  |  | OTU_340 |
|  |  | OTU_2516 |
|  |  | OTU_311 |
|  |  | OTU_413 |
|  |  | OTU_348 |
|  |  | OTU_298 |
|  |  | OTU_1308 |
|  |  | OTU_1447 |
|  |  | OTU_746 |
|  |  | OTU_1019 |
|  |  | OTU_1182 |
|  |  | OTU_2448 |
| Phylum Actinobacteria, Order Actinomycetales | | |
| Fam.Cellulomonadaceae | *Cellulomonas* | OTU_349 |
|  |  | OTU_402 |
| Fam.Streptomycetaceae | *Streptomyces* | OTU_688 |
|  |  | OTU_2679 |
| Fam.Nocardiopsaceae | *Thermobifida* | OTU_645 |
| Fam.Microbacteriaceae | *Microbacterium* | OTU_197 |
|  |  | OTU_144 |
| Phylum Bacteroidetes, Class Flavobacteriia, Order Flavobacteriales | | |
| Fam.Flavobacteriaceae | *Flavobacterium* | OTU_1240 |
|  |  | OTU_1005 |
|  |  | OTU_1895 |
|  |  | OTU_1334 |
|  |  | OTU_193 |
|  |  | OTU_129 |
|  |  | OTU_442 |
|  |  | OTU_1292 |
|  |  | OTU_2763 |
|  |  | OTU_2161 |
|  |  | OTU_1701 |
|  |  | OTU_507 |
|  |  | OTU_2788 |
|  |  | OTU_582 |
|  |  | OTU_1235 |
|  |  | OTU_613 |
|  |  | OTU_2024 |
| Fam.Weeksellaceae | *Chryseobacterium* | OTU_274 |
|  |  | OTU_431 |
|  |  | OTU_258 |
|  |  | OTU_521 |
|  |  | OTU_2147 |
|  |  | OTU_775 |
|  |  | OTU_418 |
|  |  | OTU_432 |
|  |  | OTU_1832 |
|  |  | OTU_2152 |
| Phylum Bacteroidetes, Class Sphingobacteriia, Order Sphingobacteriales | | |
| Fam.Sphingobacteriaceae | *Sphingobacterium* | OTU_60 |
|  |  | OTU_1445 |
|  |  | OTU_195 |
|  |  | OTU_35 |
|  |  | OTU_1898 |
|  |  | OTU_126 |
|  |  | OTU_1693 |
|  |  | OTU_472 |
|  |  | OTU_738 |
|  |  | OTU_385 |
|  |  | OTU_979 |
|  |  | OTU_157 |
|  |  | OTU_98 |
|  |  | OTU_138 |
|  |  | OTU_132 |
|  |  | OTU_352 |
|  |  | OTU_600 |
|  |  | OTU_1010 |
|  |  | OTU_935 |
|  |  | OTU_713 |
|  |  | OTU_1362 |
|  |  | OTU_1131 |
|  |  | OTU_1639 |
|  |  | OTU_350 |
| Phylum Proteobacteria, Class Alphaproteobacteria, Order Rhizobiales | | |
| Fam.Brucellaceae | *Ochrobactrum* | OTU_73 |
| Phylum Proteobacteria, Class Betaproteobacteria, Order Burkholderiales | | |
| Fam.Alcaligenaceae | *Achromobacter* | OTU_672 |
|  |  | OTU_317 |
|  |  | OTU_42 |
|  |  | OTU_12 |
| Phylum Proteobacteria, Class Gammaproteobacteria, Order Alteromonadales | | |
| Fam.Alteromonadaceae | *Cellvibrio* | OTU_1846 |
|  |  | OTU_843 |
|  |  | OTU_2263 |
|  |  | OTU_2416 |
|  |  | OTU_1231 |
| Phylum Proteobacteria, Class Gammaproteobacteria, Order Pseudomonadales | | |
| Fam.Pseudomonadaceae | *Pseudomonas* | OTU_1964 |
|  |  | OTU_264 |
|  |  | OTU_56 |
|  |  | OTU_103 |
|  |  | OTU_268 |
|  |  | OTU_226 |
|  |  | OTU_283 |
|  |  | OTU_308 |
|  |  | OTU_161 |
|  |  | OTU_447 |
|  |  | OTU_218 |
|  |  | OTU_185 |
|  |  | OTU_63 |
|  |  | OTU_292 |
|  |  | OTU_32 |
|  |  | OTU_508 |
|  |  | OTU_527 |
|  |  | OTU_498 |
|  |  | OTU_824 |
| Phylum Proteobacteria, Class Gammaproteobacteria, Order Enterobacteriales | | |
| Fam.Enterobacteriaceae | *Proteus* | OTU_273 |
|  |  | OTU_257 |
|  | *Enterobacter* | OTU_1149 |
|  | *Serratia* | OTU_107 |
|  |  | OTU_17 |
|  |  | OTU_8 |
|  |  | OTU_115 |
|  |  | OTU_306 |
| Phylum Proteobacteria, Class Gammaproteobacteria, Order Xanthomonadales | | |
| Fam.Xanthomonadaceae | *Stenotrophomonas* | OTU_136 |
|  |  | OTU_151 |
|  |  | OTU_362 |
|  |  | OTU_556 |

“^1^”: 131 OTUs were selected according to the genera characterized by Kong et al., 2014, Zhu et al., 2011, Bashir et al., 2013, Dantur et al., 2015 and Manfredi et al., 2015.

**Table S3. 16S rRNA sequences of cellulolytic bacteria from red panda, *Ailurus fulgens* (Kong et al., 2014)**

| **Phylogeny** | **Genus** | **Species** | **GenBank Accession Number** |
| --- | --- | --- | --- |
| Phylum Firmicutes, Class Clostridia, Order Clostridiales | | | |
| Fam. Syntrophomonodaceae | *Caldocellulosiruptor* | *saccharolyticus* | NR_036878.1 |
|  | *Caldocellulosiruptor* | *lactoaceticus* | NR_026231.1 |
|  | *Caldocellulosiruptor* | *kristjanssonii* | NR_025279.1 |
|  | *"Anaerocellum"* | *thermophilum* | L09180.1 |
| Fam. Lachnospiraceae | *Butyrivibrio* | *fibrisolvens* | EF427364.1 |
|  | *Ruminococcus* | *flavefaciens* | AM748742.1 |
|  | *Ruminococcus* | *succinogenes* | M62696.1 |
|  | *Ruminococcus* | *albus* | AF079847.1 |
| Fam. Clostridiaceae | *Acetivibrio* | *cellulolyticus* | L35516.1 |
|  | *Acetivibrio* | *cellulosolvens* CD2 | NR_025917.1 |
|  | *Acetivibrio* | *cellulosolvens* ATCC 35928 | L35515.1 |
|  | *Bacteroides* | *cellulosolvens* | L35517.1 |
|  | *Bacteroides* | sp. P-1 | JX041639.1 |
|  | *Clostridium* | *acetobutylicum* | AB675523.1 |
|  | *Clostridium* | *aldrichii* P-1 | NR_026099.2 |
|  | *Clostridium* | *cellobioparum* | NR_026104.1 |
|  | *Clostridium* | *cellulofermentans* DSM 5628 | NR_026100.1 |
|  | *Clostridium* | *cellulolyticum* | NR_102768.1 |
|  | *Clostridium* | *cellulosi* | NR_044624.1 |
|  | *Clostridium* | *chartatabidum* | NR_029239.1 |
|  | *Clostridium* | *herbivorans* | L34418.1 |
|  | *Clostridium* | *josui* | AB011057.1 |
|  | *Clostridium* | *papyrosolvens* | NR_026102.1 |
|  | *Clostridium* | *stercorarium* | L09174.1 |
|  | *Clostridium* | *thermocellum* (DSM 1237) | L09173.1 |
|  | *Clostridium* | *thermocellum* ATCC 27405 | NR_074629.1 |
|  | *Clostridium* | *thermocopriae* JT-3 | NR_025898.1 |
| Phylum Actinobacteria, Order Actinomycetales | | | |
| Subord. Frankineae, Fam. Acidothermaceae | *Acidothermus* | *cellulolyticus* | AJ007290.1 |
| Subord. Micromonosporineae, Fam. Cellulomonadaceae | *Cellulomonas* | *biazotea* | X83802.1 |
|  | *Cellulomonas* | *cellasea* | X83804.1 |
|  | *Cellulomonas* | *cellulans* | X83809.1 |
|  | *Cellulomonas* | *fimi* | NR_074509.1 |
|  | *Cellulomonas* | *flavigena* | X83799.1 |
|  | *Cellulomonas* | *gelida* | X83800.1 |
|  | *Cellulomonas* | *iranensis* | NR_024914.1 |
|  | *Cellulomonas* | *persica* | AF064701.1 |
|  | *Cellulomonas* | *uda* | X83801.1 |
| Fam. Micromonosporaceae | *Streptomyces* | *reticuli* | GU383165.1 |
| Subord. Streptomycineae,  Fam. Streptomycetaceae | *Streptomyces* | *aureofaciens* | EF017714.1 |
|  | *Streptomyces* | *flavogriseus* | NR_074559.1 |
|  | *Streptomyces* | *lividans* | X86354.1 |
|  | *Streptomyces* | *nitrosporeus* | HQ439420.1 |
|  | *Streptomyces* | *rochei* | KC522303.1 |
|  | *Streptomyces* | *thermovulgaris* | Z68094.1 |
| Subord. Streptosporangiaceae,  Fam. Nocardiopsaceae | *Microbispora* | *bispora* ATCC19993 | U58523.1 |
|  | *Microbispora* | *bispora* JCM3082 | U58524.1 |
|  | *Thermobifida* | *alba* | AB304877.1 |
|  | *Thermobifida* (*Thermomonospora*) | *fusca* | AM932257.1 |
|  | *Thermomonospora* | *curvata* | X97893.1 |
| Phylum Fibrobacteres, Class Fibrobacteres, Order Fibrobacterales | | | |
| Fam. Fibrobacteriaceae | *Fibrobacter* | *succinogenes* S85 | AJ496032.1 |
| Phylum Bacteroidetes, Class Sphingobacteria, Order Sphingobacteriales | | | |
| Fam. Flexibacteriaceae | *Sporocytophaga* | *myxococcoides* DSM 11118 | NR_025463.1 |
|  | *Sporocytophaga* | *myxococcoides* | AB681028.1 |
| Phylum Bacteroidetes, Class Flavobacteria, Order Flavobacteriales | | | |
| Fam. Flavobacteriaceae | *Flavobacterium* | *johnsoniae* | NR_044738.1 |
| Phylum Proteobacteria, Class Gammaproteobacteria, Order Pseudomonadales | | | |
| Fam. Pseudomonaceae | *Cellvibrio* | *fulvus* | NR_025210.1 |
|  | *Cellvibrio* | *gilvus* | NR_074443.1 |
|  | *Cellvibrio* | *mixtus* | KC329916.1 |
|  | *Cellvibrio* | *vulgaris* | NR_025209.1 |
|  | *Pseudomonas* | *fluorescens* (*cellulosa*) | DQ282185.1 |
|  | *Pseudomonas* | *mendocina* | M59154.1 |

**Table S4. The microbial flora of wild and captive pandas (Zhu et al., 2011)**

| **OUT ID** | **Phylum** | **GenBank Accession Number** |
| --- | --- | --- |
| OTU1 Zhu | Proteobacteria | JF920308 |
| OTU2 Zhu | Proteobacteria | JF920309 |
| OTU3 Zhu | Proteobacteria | JF920310 |
| OTU4 Zhu | Proteobacteria | JF920311 |
| OTU5 Zhu | Proteobacteria | JF920312 |
| OTU6 Zhu | Proteobacteria | JF920313 |
| OTU7 Zhu | Proteobacteria | JF920314 |
| OTU8 Zhu | Proteobacteria | JF920315 |
| OTU9 Zhu | Proteobacteria | JF920316 |
| OTU10 Zhu | Proteobacteria | JF920317 |
| OTU11 Zhu | Proteobacteria | JF920318 |
| OTU12 Zhu | Proteobacteria | JF920319 |
| OTU13 Zhu | Firmicutes-Bacilli | JF920320 |
| OTU14 Zhu | Firmicutes-Bacilli | JF920321 |
| OTU15 Zhu | Firmicutes-Bacilli | JF920322 |
| OTU16 Zhu | Firmicutes-Bacilli | JF920323 |
| OTU17 Zhu | Firmicutes-Bacilli | JF920324 |
| OTU18 Zhu | Firmicutes-Bacilli | JF920325 |
| OTU19 Zhu | Firmicutes-Bacilli | JF920326 |
| OTU20 Zhu | Firmicutes-Bacilli | JF920327 |
| OTU21 Zhu | Firmicutes-Bacilli | JF920328 |
| OTU22 Zhu | Firmicutes-Bacilli | JF920329 |
| OTU23 Zhu | Firmicutes-Bacilli | JF920330 |
| OTU24 Zhu | Firmicutes-Bacilli | JF920331 |
| OTU25 Zhu | Firmicutes-Bacilli | JF920332 |
| OTU26 Zhu | Firmicutes-Bacilli | JF920333 |
| OTU27 Zhu | Firmicutes-Bacilli | JF920334 |
| OTU28 Zhu | Firmicutes-Bacilli | JF920335 |
| OTU29 Zhu | Firmicutes-Bacilli | JF920336 |
| OTU30 Zhu | Firmicutes-Bacilli | JF920337 |
| OTU31 Zhu | Firmicutes-Bacilli | JF920338 |
| OTU32 Zhu | Firmicutes-Bacilli | JF920339 |
| OTU33 Zhu | Firmicutes-Bacilli | JF920340 |
| OTU34 Zhu | Firmicutes-Bacilli | JF920341 |
| OTU35 Zhu | Firmicutes-Bacilli | JF920342 |
| OTU36 Zhu | Firmicutes-Bacilli | JF920343 |
| OTU37 Zhu | Firmicutes-Bacilli | JF920344 |
| OTU38 Zhu | Firmicutes-Bacilli | JF920345 |
| OTU39 Zhu | Firmicutes-Bacilli | JF920346 |
| OTU40 Zhu | Firmicutes-Bacilli | JF920347 |
| OTU41 Zhu | Firmicutes-Bacilli | JF920348 |
| OTU42 Zhu | Firmicutes-Clostridia | JF920349 |
| OTU43 Zhu | Firmicutes-Clostridia | JF920350 |
| OTU44 Zhu | Firmicutes-Clostridia | JF920351 |
| OTU45 Zhu | Firmicutes-Clostridia | JF920352 |
| OTU46 Zhu | Firmicutes-Clostridia | JF920353 |
| OTU47 Zhu | Firmicutes-Clostridia | JF920354 |
| OTU48 Zhu | Firmicutes-Clostridia | JF920355 |
| OTU49 Zhu | Firmicutes-Clostridia | JF920356 |
| OTU50 Zhu | Firmicutes-Clostridia | JF920357 |
| OTU51 Zhu | Firmicutes-Clostridia | JF920358 |
| OTU52 Zhu | Firmicutes-Clostridia | JF920359 |
| OTU53 Zhu | Firmicutes-Clostridia | JF920360 |
| OTU54 Zhu | Firmicutes-Clostridia | JF920361 |
| OTU55 Zhu | Firmicutes-Clostridia | JF920362 |
| OTU56 Zhu | Firmicutes-Clostridia | JF920363 |
| OTU57 Zhu | Firmicutes-Clostridia | JF920364 |
| OTU58 Zhu | Firmicutes-Clostridia | JF920365 |
| OTU59 Zhu | Firmicutes-Clostridia | JF920366 |
| OTU60 Zhu | Firmicutes-Clostridia | JF920367 |
| OTU61 Zhu | Firmicutes-Clostridia | JF920368 |
| OTU62 Zhu | Firmicutes-Clostridia | JF920369 |
| OTU63 Zhu | Firmicutes-Clostridia | JF920370 |
| OTU64 Zhu | Firmicutes-Clostridia | JF920371 |
| OTU65 Zhu | Firmicutes-Clostridia | JF920372 |
| OTU66 Zhu | Firmicutes-Clostridia | JF920373 |
| OTU67 Zhu | Firmicutes-Clostridia | JF920374 |
| OTU68 Zhu | Firmicutes-Clostridia | JF920375 |
| OTU69 Zhu | Firmicutes-Clostridia | JF920376 |
| OTU70 Zhu | Firmicutes-Clostridia | JF920377 |
| OTU71 Zhu | Firmicutes-Clostridia | JF920378 |
| OTU72 Zhu | Firmicutes-Clostridia | JF920379 |
| OTU73 Zhu | Firmicutes-Clostridia | JF920380 |
| OTU74 Zhu | Firmicutes-Clostridia | JF920381 |
| OTU75 Zhu | Cyanobacteria | JF920382 |
| OTU76 Zhu | Acidobacteria | JF920383 |
| OTU77 Zhu | Acidobacteria | JF920384 |
| OTU78 Zhu | Acidobacteria | JF920385 |
| OTU79 Zhu | Acidobacteria | JF920386 |
| OTU80 Zhu | Acidobacteria | JF920387 |
| OTU81 Zhu | Acidobacteria | JF920388 |
| OTU82 Zhu | Acidobacteria | JF920389 |
| OTU83 Zhu | Acidobacteria | JF920390 |
| OTU84 Zhu | Cyanobacteria | JF920391 |
| OTU85 Zhu | Bacteroidetes | JF920392 |

**Table S5. 16S rRNA sequences of cellulolytic bacteria from other sources (Bashir et al., 2013; Dantur et al., 2015; Manfredi et al., 2015)**

| **Nearest relative** | **GenBank Accession Number** |
| --- | --- |
| ***Termite, pill-bug and stem-borer guts (Bashir et al., 2013)*** | |
| *Bacillus* sp. 6063 | KC434960 |
| *Klebsiella* sp. clone F7 | KC434961 |
| *Trabulsiella guamensis* GTC1379 | KC434962 |
| *Bacillus pumilus* BSH4 | KC434963 |
| *Bacillus* sp. SCSSS10 | KC434964 |
| *Pantoea agglomerans* WAB1927 | KC434965 |
| *Bacillus licheniformis* EdyKolBl23 | KC434966 |
| *Bacillus licheniformis* BCRC 15413 | KC434967 |
| *Bacillus licheniformis* SubaMucBl16 | KC434968 |
| *Bacillus cereus* TAUC5 | KC434969 |
| *Bacillus cereus* F837/76 | KC434970 |
| *Bacillus subtilis* K21 | KC434971 |
| *Paenibacillus polymyxa* DSM 36T | KC434972 |
| *Paenibacillus polymyxa* YRL13 | KC434973 |
| *Bacillus subtilis* M50 | KC434974 |
| *Bacillus subtilis* M16K | KC434975 |
| *Enterobacter aerogenes* KCTC 2190 | KC434976 |
| *Cellulosimicrobium* sp. TUT1242 | KC434977 |
| *Bacillus* species BB2_1A | KC434978 |
| *Bacillus subtilis* AQ1 | KC434979 |
| *Microbacteriaceae bacterium* HLB-6 | KC434980 |
| *Microbacteriaceae bacterium* BMC-3 | KC434981 |
| *Microbacterium oleivorans* CCGE2277 | KC434982 |
| *Microbacterium arborescens* DSM 20754 | KC434983 |
| *Microbacterium arborescens* DSM 20754 | KC434984 |
| *Microbacterium arborescens* JB8_2B | KC434985 |
| *Klebsiella pneumoniae* L-13 | KC434996 |
| *Klebsiella pneumoniae* L-13 | KC434997 |
| Microbacteriaceae bacterium BMC-3 | KC434998 |
| *Bacillus subtilis* C1Y001 | KC434999 |
| Enterobacter species DHM1T | KC43500 |
| *Serratia marcescens* PS1 | KC43501 |
| *Bacillus* sp. PR 1.7 | KC434993 |
| *Pantoea species* NCCP116 | KC434994 |
| *Pantoea agglomerans* WAB1927 | KC434995 |
| *Bacillus subtilis* KL-073 | KC434990 |
| *Bacillus thuringiensis* 2PR56-10 | KC434991 |
| *Bacillus tequilensis* VITJAAM2 | KC434992 |
| *Bacillus subtilis* M50 | KC434986 |
| *Bacillus licheniformis* ACO1 | KC434987 |
| *Trabulsiella guamensis* GTC1379 | KC434988 |
| *Bacillus* sp. DV9-35 | KC434989 |
| ***The sugarcane borer, Diatraea saccharalis, larvae guts (Dantur et al., 2015)*** | |
| *Klebsiella oxytoca* | KM096608/DSM 27019 |
| *Klebsiella pneumoniae* | KM096599 |
| *Klebsiella variicola* | KM096598/DSM 27017 |
| *Stenotrophomonas maltophilia* | KM096600 |
| *Stenotrophomonas rhizophila* | KM096602 |
| ***Industrial samples and the gut of native insects from Northwest of Argentina (Manfredi et al., 2015)*** | |
| *Bacillus tequilensis* KCTC 13622(T) | LN829557 |
| *Bacillus subtilis* subsp. *subtilis* NCIB 3610(T) | LN829558 |
| *Bacillus anthracis* ATCC 14578(T) | LN829559 |
| *Bacillus sonorensis* NBRC 101234(T) | LN829560 |
| *Bacillus tequilensis* KCTC 13622(T) | LN829561 |
| *Bacillus cereus* ATCC 14579(T) | LN829562 |
| *Bacillus firmus* NCIMB 9366(T) | LN829563 |
| *Bacillus sonorensis* NBRC 101234(T) | LN829564 |
| *Bacillus tequilensis* KCTC 13622(T) | LN829565 |
| *Bacillus siamensis* KCTC 13613(T) | LN829566 |
| *Bacillus cereus* ATCC 14579(T) | LN829567 |
| *Bacillus amyloliquefaciens* subsp. *plantarum* FZB42(T) | LN829568 |
| *Bacillus safensis* FO‐36b(T) | LN829569 |
| *Bacillus tequilensis* KCTC 13622(T) | LN829570 |
| *Bacillus safensis* FO‐36b(T) | LN829571 |
| *Bacillus anthracis* ATCC 14578(T) | LN829572 |
| *Bacillus anthracis* ATCC 14578(T) | LN829573 |
| *Bacillus siamensis* KCTC 13613(T) | LN829574 |
| *Bacillus siamensis* KCTC 13613(T) | LN829575 |
| *Bacillus amyloliquefaciens* subsp. *plantarum* FZB42(T) | LN829576 |
| *Paenibacillus cineris* LMG 18439(T) | LN829577 |
| *Paenibacillus lautus* NRRL NRS‐666(T) | LN829578 |
| *Paenibacillus cineris* LMG 18439(T) | LN829579 |
| *Paenibacillus cineris* LMG 18439(T) | LN829580 |
| *Cohnella formosensis* CC‐Alfalfa‐35(T) | FJ976043.1 |
| *Brevibacillus parabrevis* IFO 12334(T) | LN829581 |
| *Staphylococcus warneri* ATCC 27836(T) | LN829582 |
| *Staphylococcus warneri* ATCC 27836(T) | LN829583 |
| *Microbacterium paraoxydans* CF36(T) | LN832405 |
| *Microbacterium paraoxydans* CF36(T) | LN829584 |
| *Agromyces mediolanus* DSM 20152(T) | LN829585 |
| *Pseudomonas aeruginosa* JCM 5962(T) | LN832407 |
| *Pseudomonas aeruginosa* JCM 5962(T) | LN829586 |
| *Pseudomonas hibiscicola* ATCC 19867(T) | LN829587 |
| *Pseudomonas stutzeri* ATCC 17588(T) | LN829588 |
| *Pseudomonas plecoglossicida* FPC951(T) | LN829589 |
| *Pseudomonas guguanensis* CC‐G9A(T) | LN829590 |
| *Pseudomonas aeruginosa* JCM 5962(T) | LN829591 |
| *Pseudomonas aeruginosa* JCM 5962(T) | LN829592 |
| *Enterobacter cloacae subsp. dissolvens* LMG 2683(T) | LN829595 |
| *Achromobacter insolitus* LMG 6003(T) | LN829593 |
| *Achromobacter insolitus* LMG 6003(T) | LN829594 |
| *Chryseobacterium contaminans* C26(T) | LN829596 |
| *Chryseobacterium contaminans* C26(T) | LN832406 |
| *Sphingobacterium multivorum* IAM14316(T) | LN829597 |

**Table S6.** **Number of gut bacterial sequences from each insect sample**

| **Sample number** | **Sample ID** | **Insect species** | **High-quality sequence reads after normalization** |
| --- | --- | --- | --- |
| 1 | CNM1 | *Ceracris nigricornis*  (Male) | 13680 |
| 2 | CNM2 |  | 2922 |
| 3 | CNM3 |  | 2156 |
| 4 | CNM4 |  | 7912 |
| 5 | CNM5 |  | 9167 |
| 6 | CNM6 |  | 120526 |
| 7 | CNM7 |  | 47261 |
| 8 | CNM8 |  | 5351 |
| 9 | CNF1 | *Ceracris nigricornis*  (Female) | 5042 |
| 10 | CNF2 |  | 4037 |
| 11 | CNF3 |  | 17281 |
| 12 | CNF4 |  | 8890 |
| 13 | CNF5 |  | 11363 |
| 14 | CNF6 |  | 4785 |
| 15 | CNF7 |  | 4130 |
| 16 | CNF8 |  | 5527 |
| 17 | CC1 | *Crypsiptya coclesalis* | 4816 |
| 18 | CC2 |  | 2031 |
| 19 | CC3 |  | 1651 |
| 20 | CC4 |  | 13292 |
| 21 | CC5 |  | 2025 |
| 22 | CC6 |  | 50774 |
| 23 | CC7 |  | 9921 |
| 24 | CC8 |  | 5109 |
| 25 | SE1 | *Sinibotys evenoralis* | 11931 |
| 26 | SE2 |  | 11161 |
| 27 | SE3 |  | 21923 |
| 28 | SE4 |  | 6512 |
| 29 | SE5 |  | 4875 |
| 30 | SE6 |  | 7539 |
| 31 | SE7 |  | 4163 |
| 32 | SE8 |  | 7632 |
| 33 | MC1 | *Melanotus cribricollis* | 20125 |
| 34 | MC2 |  | 28981 |
| 35 | MC3 |  | 23675 |
| 36 | MC4 |  | 34931 |
| 37 | MC5 |  | 42297 |
| 38 | MC6 |  | 25189 |
| 39 | MC7 |  | 5154 |
| 40 | MC8 |  | 50829 |
| 41 | AK1 | *Apamea kumaso* | 28480 |
| 42 | AK2 |  | 13139 |
| 43 | AK3 |  | 13910 |
| 44 | AK4 |  | 7097 |
| 45 | AK5 |  | 8976 |
| 46 | AK6 |  | 11454 |
| 47 | AK7 |  | 16620 |
| 48 | AK8 |  | 13084 |
| 49 | CB1 | *Cyrtotrachelus buqueti* | 47638 |
| 50 | CB2 |  | 61996 |
| 51 | CB3 |  | 21341 |
| 52 | CB4 |  | 33625 |
| 53 | CB5 |  | 25600 |
| 54 | CB6 |  | 52793 |
| 55 | CB7 |  | 10070 |
| 56 | CB8 |  | 16933 |
| 57 | CT1 | *Cyrtotrachelus thompsoni* | 25917 |
| 58 | CT2 |  | 32419 |
| 59 | CT3 |  | 10034 |
| 60 | CT4 |  | 15707 |
| 61 | PB1 | *Pseudoregma bambucicola* | 671354 |
| 62 | PB2 |  | 260463 |
| 63 | PB3 |  | 440097 |
| 64 | PB4 |  | 736354 |
| 65 | PB5 |  | 443426 |
| 66 | PB6 |  | 514756 |
| 67 | PB7 |  | 953844 |
| 68 | PB8 |  | 661796 |
| 69 | HD1 | *Hippotiscus dorsalis* | 282081 |
| 70 | HD2 |  | 42767 |
| 71 | HD3 |  | 1043566 |
| 72 | HD4 |  | 923332 |
| 73 | HD5 |  | 400096 |
| 74 | HD6 |  | 48055 |
| 75 | HD7 |  | 60298 |
| 76 | HD8 |  | 1469871 |

**Table S7.** **Anosim analysis of gut bacteria communities under different treatments**

| **Treatments** | **Insect species^1^** | | **Gut bacteria** | | **Cellulolytic bacteria** | |
| --- | --- | --- | --- | --- | --- | --- |
|  |  |  | **R^2^** | **P^3^** | **R** | **P** |
| **Species** | Leaf | CC *vs* SE | -0.0039 | **0.378** | -0.0151 | **0.464** |
|  |  | CC *vs* CNM | 0.5106 | **0.002** | 0.6300 | **0.001** |
|  |  | CC *vs* CNF | 0.6200 | **0.001** | 0.2316 | **0.016** |
|  |  | SE *vs* CNM | 0.4492 | **0.001** | 0.5681 | **0.001** |
|  |  | SE *vs* CNF | 0.5039 | **0.001** | 0.4425 | **0.002** |
|  |  | CNM *vs* CNF | 0.4604 | **0.002** | 0.6925 | **0.002** |
|  |  | CC *vs* SE *vs* CNM *vs* CNF | 0.4209 | **0.001** | 0.4267 | **0.001** |
|  | Shoot | MC *vs* AK | 0.6110 | **0.001** | 0.6959 | **0.003** |
|  |  | MC *vs* CB | 0.8700 | **0.001** | 0.5246 | **0.002** |
|  |  | AK *vs* CB | 0.8990 | **0.001** | 0.5262 | **0.001** |
|  |  | MC *vs* CT | 0.7812 | **0.003** | 0.3548 | **0.035** |
|  |  | AK *vs* CT | 0.9026 | **0.004** | 0.421 | **0.017** |
|  |  | CB *vs* CT | -0.1507 | **0.820** | -0.1287 | **0.806** |
|  |  | MC *vs* AK *vs* CB *vs* CT | 0.6831 | **0.001** | 0.4502 | **0.001** |
|  | Sap | PB *vs* HD | 1.0000 | **0.001** | – | – |
| **Orders** | Lepidoptera *vs* Orthoptera | | 0.2941 | **0.001** | 0.1828 | **0.008** |
|  | Lepidoptera *vs* Coleoptera | | 0.5659 | **0.001** | 0.3992 | **0.001** |
|  | Coleoptera *vs* Orthoptera | | 0.4879 | **0.001** | 0.4232 | **0.001** |
|  | Lepidoptera *vs* Orthoptera vs Coleoptera | | 0.4529 | **0.001** | 0.3425 | **0.001** |
|  | Coleoptera *vs* Hemiptera | | 0.9171 | **0.001** | – | – |
|  | Lepidoptera *vs* Hemiptera | | 0.9172 | **0.001** | – | – |
|  | Orthoptera *vs* Hemiptera | | 0.8457 | **0.001** | – | – |
|  | Lepidoptera *vs* Orthoptera vs Coleoptera *vs* Hemiptera | | 0.6213 | **0.001** | – | – |
| **Diets** | Leaf *vs* Shoot | | 0.4541 | **0.001** | 0.2442 | **0.001** |
|  | Leaf *vs* Sap | | 0.9447 | **0.001** | – | – |
|  | Shoot *vs* Sap | | 0.9429 | **0.001** | – | – |
|  | Leaf *vs* Shoot *vs* Sap | | 0.6855 | **0.001** | – | – |

“^1^”: Insect species include male and female of *Ceracris nigricornis* (CNM & CNF), *Crypsiptya coclesalis* (CC), *Sinibotys evenoralis* (SE), *Melanotus cribricollis* (MC), *Apamea kumaso* (AK), *Cyrtotrachelus buqueti* (CB), *Cyrtotrachelus thompsoni* (CT), *Pseudoregma bambucicola* (PB), and *Hippotiscus dorsalis* (HD).

“^2^”: R>0: Difference between groups is greater than difference within group.

“^3^”: P<0.05: Significant difference between groups (bolded). P>0.05 is highlighted in red.
